# Regional surveillance of carbapenem-resistant *Klebsiella pneumoniae* responsible for bloodstream infections in Hebei province

**DOI:** 10.3389/fcimb.2026.1883044

**Published:** 2026-08-14

**Authors:** Yuyao Hao, Jianhui Li, Junxian Zhang, Shoujun Xie, Mingzhen Zhao, Wei Zhang, Li Xu, Hongtao Ren, Hongmeng Ji, Xueshan Sun, Jidong Zhang, Hongxia Zhang, Hainan Wen

**Affiliations:** 1 Department of Clinical Laboratory, The Affiliated Hospital of Chengde Medical University, Chengde, Hebei, China; 2 Department of Preventive Medicine, Chengde Medical University, Chengde, Hebei, China; 3 Hebei Key Laboratory of Panvascular Diseases, Chengde, Hebei, China; 4 Hebei Key Laboratory of Pathogenic Mechanisms and Diagnosis & Treatment Technologies for Lung Microbiome, Zhangjiakou, Hebei, China; 5 The First Affiliated Hospital of Hebei North University, Zhangjiakou, Hebei, China; 6 Department of Clinical Laboratory, Cangzhou Central Hospital, Cangzhou, Hebei, China; 7 Department of Clinical Laboratory, Xingtai People’s Hospital, Xingtai, Hebei, China; 8 Department of Clinical Laboratory, Cangzhou Hospital of Integrated Traditional Chinese and Western Medicine, Cangzhou, Hebei, China; 9 Department of Clinical Laboratory, Baoding NO.1 Hospital, Baoding, Hebei, China; 10 Department of Clinical Laboratory, People’s Hospital of Zunhua City, Zunhua, Tangshan, Hebei, China; 11 Department of Clinical Laboratory, Gucheng County Hospital of Hebei Province, Hengshui, Hebei, China

**Keywords:** antimicrobial susceptibility, bloodstream infection, carbapenem-resistant *Klebsiella pneumoniae*, hypervirulence, molecular epidemiological characteristics

## Abstract

**Objective:**

To investigate the epidemiological characteristics of CRKP responsible for BSIs in Hebei Province, China.

**Methods:**

A total of 100 CRKP isolates were obtained across 15 hospitals in eight cities in Hebei Province, China, between 2022 and 2024. Polymerase chain reaction and short-read whole-genome sequencing were used to characterize the molecular profiles of the strains.

**Results:**

All isolates exhibited high-level resistance to β-lactam antibiotics, including a 100% resistance rate to carbapenems and resistance rates of > 97% to β-lactamase inhibitor combinations. The *blaKPC* was the predominant carbapenemase gene (89%, 89/100). In this study, 62 carbapenem-resistant *Klebsiella pneumoniae* isolates harboring multiple hypervirulence-related genes (CR-hvKp) and 38 carbapenem-resistant classical *Klebsiella pneumoniae* (CR-cKp) isolates were identified. The predominant plasmid replicon types were IncFII(pHN7A8) (69%, 69/100) and ColRNAI (71%, 71/100). Notably, according to the core-genome SNP analysis, the ST11-KL64 subclone was the predominant lineage among the sampled isolates (48%, 48/100).

**Conclusions:**

The CRKP isolates from BSIs exhibit significant drug resistance and high virulence characteristics, warranting widespread clinical attention.

## Introduction

Bloodstream infections (BSIs) constitute an important global health challenge, characterized by a high prevalence and potentially fatal outcomes (Wisplinghoff et al., 2004; Marra et al., 2011; Diekema et al., 2019; Kontula et al., 2021; Verway et al., 2022). With an estimated annual incidence of 150 cases per 100,000 individuals and a crude 30-day mortality rate of 17%, BSIs are a major contributor to patient morbidity and mortality (Verway et al., 2022) and represent the second leading cause of death attributable to antimicrobial resistance, after respiratory tract infections (Antimicrobial RC, 2022). Among the various pathogens responsible for BSIs, *Klebsiella pneumoniae* consistently ranks as the third most common etiological agent after *Escherichia coli* and *Staphylococcus aureus* (Diekema et al., 2019), and recent evidence indicates a particularly high mortality rate of 43.0% for BSIs specifically caused by *K. pneumoniae* (Yang et al., 2024).

*K. pneumoniae* is an opportunistic pathogen capable of inducing severe systemic illness. Contemporary taxonomy delineates two primary pathotypes based on phenotypic and genotypic profiles: classical *K. pneumoniae* (cKp) and hypervirulent *K. pneumoniae* (hvKp) (Podschun and Ullmann, 1998; Russo and Marr, 2019). cKp strains are typically associated with multidrug resistance (MDR) but exhibit comparatively lower virulence, predominantly causing nosocomial infections in settings such as hospitals and long-term care facilities, as documented in North America and Europe (Moellering, 2010; Snitkin et al., 2012; Conlan et al., 2014; Dong et al., 2022; Pu et al., 2023). Conversely, hvKp strains, while often retaining antimicrobial susceptibility, demonstrate enhanced virulence factors leading to severe community-acquired infections (Russo and Marr, 2019; Pu et al., 2023).

The increasing use of broad-spectrum antibiotics has driven the emergence and dissemination of MDR *K. pneumoniae*, with carbapenem-resistant strains posing a particularly critical challenge to clinical management and public health (Meatherall et al., 2009; Navon-Venezia et al., 2017). The World Health Organization (WHO) has accordingly prioritized third-generation cephalosporin-resistant and carbapenem-resistant Enterobacteriaceae, including *K. pneumoniae*, as pathogens of critical concern (Tacconelli et al., 2018). Mortality rates for BSIs caused by resistant *K. pneumoniae* remain alarmingly high, ranging from 17%–34% for extended-spectrum β-lactamase-producing strains to as high as 42% for carbapenem-resistant *K. pneumoniae* (CRKP) (Xu et al., 2017; Scheuerman et al., 2018; Sianipar et al., 2019; Li et al., 2023; Paniagua-García et al., 2024). CRKP isolates account for approximately 90% of all clinical carbapenem-resistant Enterobacteriaceae infections in China (Zhang et al., 2017). Tian et al. integrated epidemiological and genomic data and showed that ST11 and ST307 Kp isolates were associated with nosocomial outbreak and transmission. Comparative analysis of 147 *Klebsiella pneumoniae* genomes and 39 fully assembled chromosomes revealed that all ST11 Kp isolates carrying the *blaKPC-2* carbapenemase gene exhibited reduced susceptibility to tigecycline (Yang et al., 2021). Chen et al. indicated, through phylogenetic and SNP analysis, that the transmission of CRKP strains in China likely involved several major clones of ST11 (Yang et al., 2022). Research findings from He et al. showed that expansion of CRKP capsule type KL64 (59.5%) alongside decreases in KL47 prevalence. Phylogenetic and spatiotemporal analysis revealed differential geographical prevalence of KL47 and KL64 strains across China (Hu et al., 2024). A nationwide molecular epidemiological study by Wang et al. revealed that CRKP in China was predominantly characterized by the *blaKPC-2* carbapenemase gene, with significant geographical clustering of the ST11 clone and KL64 emerging as a dominant strain (Wang et al., 2024). Notably, their analysis of 1,631 ST11 CRKP-infected patients indicated that the associated BSIs were associated with the highest 30-day mortality rate (35.2%) among all infection types (Wang et al., 2024).

Despite these insights, detailed epidemiological data on the specific CRKP isolates responsible for BSIs, particularly within the regional context of Hebei Province, China, remain limited. We aimed to address this knowledge gap by characterizing the genomic epidemiology of CRKP strains responsible for BSIs in Hebei Province. The findings are expected to inform the development of targeted prevention strategies and support the clinical management of CRKP-associated BSIs.

## Materials and methods

### Clinical samples and bacterial isolates

A total of 100 non-repetitive CRKP isolates from patients with BSIs were collected consecutively from 15 hospitals across eight cities in Hebei Province between January 2022 and December 2024 (only one non-repetitive isolate per patient was enrolled to eliminate sampling bias). The inclusion criteria for isolates were as follows: strains recovered from patients with clinically confirmed CRKP bloodstream infections among individuals suspected of bloodstream infection. (All bloodstream infection carbapenem-resistant *Klebsiella pneumoniae* isolates from the participating hospital were consecutively enrolled in this study). All isolates were identified by matrix-assisted laser desorption/ionization time-of-flight mass spectrometry (BioMérieux, France). According to the criteria established by Lam et al. (2021), we operationally defined hvKp as *K. pneumoniae* isolates harboring hypervirulent genotypes, given that consistent diagnostic criteria for hvKp are lacking across current studies. Specifically, hvKp strains were characterized by a virulence score ≥ 3 (indicating the presence of one or more of the virulence determinants aerobactin, yersiniabactin, and/or colibactin/genotoxin) and the presence of the *rmpA* and/or *rmpA2* gene(s).

### Antimicrobial susceptibility testing

Routine antimicrobial susceptibility testing was performed using the VITEK 2 Compact automated bacterial identification system and the accompanying GN09 antimicrobial susceptibility card (BioMérieux). The results were interpreted in accordance with the Clinical and Laboratory Standards Institute guidelines M100-S35 (CLSI, 2025). Polymyxin B susceptibility was determined by the broth microdilution method, with results interpreted following the breakpoints defined by the European Committee on Antimicrobial Susceptibility Testing (EUCAST) (EUCAST, 2026). To ensure comprehensive characterization of carbapenemase production, we implemented a dual phenotypic confirmation system consisting of the modified carbapenem inactivation method (mCIM) combined with the EDTA-carbapenem inactivation method (eCIM) (CLSI, 2025).

### String test

We conducted a string test to determine if the isolated strains of CRKP possessed the hypermucoviscous phenotype (Li et al., 2021). In brief, all clinical isolates were inoculated onto blood/enteric bacterial separation agar plates and incubated at 37 °C overnight. The filaments were then stretched using disposable inoculation loops. A positive result was defined as the formation of a viscous string >5 mm long.

### Polymerase chain reaction (PCR)

Genomic DNA was extracted via the boiling lysis method (Am et al., 2025). PCR amplification was performed following protocols described in previous literature (Russo et al., 2018). The PCR reaction system for the detection of candidate biomarker genes was prepared as follows (per single reaction): 12.5 µL 2× FidCycle PCR Mix (containing Blue dye) (BBI Solutions, Sangon Biotech, Shanghai, China), 1 µL forward primer, 1 µL reverse primer (0.4 pmol/µL), 1 µL genomic DNA template (20 ng/µL), and 9.5 µL ddH_2_O. PCR was performed using an Applied Biosystems GeneAmp PCR system 9700 instrument with the following cycling conditions: step 1, 95.0 °C for 2 min; step 2, 95.0 °C for 30 s; step 3, primer-specific annealing temperature for 30 s (**Table 1**); step 4, 72 °C for 1 min; step 5, repeat steps 2 to 4 for 24 cycles; step 6, 72.0 °C for 10 min; step 7, hold at 4 °C. PCRs were resolved on a 2% agarose gel. As part of the initial development of PCR assays for biomarkers, amplification products were confirmed to contain the correct DNA sequence. Subsequently, a gene was considered present if the band of the predicted size was detected. Positive PCR amplicons were sequenced, and the results were compared with reference sequences available in GenBank (https://www.ncbi.nlm.nih.gov/blast/).

**Table 1:** Oligonucleotide primers for multiplex PCR.

Primers	Sequence (5′-3′)	Size	Temperature
*rmpA*-F	ACTGGGCTACCTCTGCTTCA	535bp	54°C
*rmpA*-R	CTTGCATGAGCCATCTTTCA		
*rmpA2*-F	CTTTATGTGCAATAAGGATGTT	447bp	55°C
*rmpA2*-R	CCTCCTGGAGAGTAAGCATT		
*iucA*-F	CTCTTCCCGCTCGCTATACT	116bp	55°C
*iucA*-R	GCATTCCACGCTTCACTTCT		
*iroB*-F	CCGCAAAGAGACGAACCGCCTT	546bp	70°C
*iroB*-R	CGGGCAATCCCCGCTTTGACTT		
*peg-344*-F	CTTGAAACTATCCCTCCAGTC	508bp	53°C
*peg-344*-R	CCAGCGAAAGAATAACCCC		
*wzi*-F	CGAGCGCTTTCTATCTTGGT	580bp	55°C
*wzi*-R	GAGAGCCACTGGTTCCAGAA		
*blaNDM*-F	ATGGAATTGCCCAATATTATGC	813bp	56°C
*blaNDM*-R	TCAGCGCAGCTTGTCGG		
*blaKPC*-F	ATGTCACTGTATCGCCGTCT	893bp	56°C
*blaKPC*-R	TTTTCAGAGCCTTACTGCCC		
*blaOXA*-F	GCGTGGTTAAGGATGAACAC	438bp	54°C
*blaOXA*-R	CATCAAGTTCAACCCAACCG		
*blaIMP*-F	TGAGCAAGTTATCTGTATTC	740bp	51°C
*blaIMP*-R	TTAGTTGCTTGGTTTTGATG		
*blaVIM*-F	TTATGGAGCAGCAACGATGT	920bp	55°C
*blaVIM*-R	CAAAAGTCCCGCTCCAACGA		
*rpoB*-F	GGCGAAATGGCWGAGAACCA	1076bp	60°C
*rpoB*-R	GAGTCTTCGAAGTTGTAACC		
*tonB*-F	CTTTATACCTCGGTACATCAGGTT	540bp	60°C
*tonB*-R	ATTCGCCGGCTGRGCRGAGAG		
*gapA*-F	TGAAATATGACTCCACTCACGG	663bp	60°C
*gapA*-R	CTTCAGAAGCGGCTTTGATGGCTT		
*infB*-F	CTCGCTGCTGGACTATATTCG	463bp	60°C
*infB*-R	CGCTTTCAGCTCAAGAACTTC		
*mdh*-F	CCCAACTCGCTTCAGGTTCAG	757bp	60°C
*mdh*-R	CCGTTTTTCCCCAGCAGCAG		
*pgi*-F	GAGAAAAACCTGCCTGTACTGCTGGC	718bp	60°C
*pgi*-R	CGCGCCACGCTTTATAGCGGTTAAT		
*phoE*-F	ACCTACCGCAACACCGACTTCTTCGG	603bp	60°C
*phoE*-R	TGATCAGAACTGGTAGGTGAT		

Concentration of oligonucleotides in final reaction (μM).

### Whole genome sequencing (WGS)

We conducted short read sequencing using 100 samples of *K. pneumoniae*. DNA was extracted using a MagPure Bacterial DNA Kit (D6361-02, Magen, China) and the concentration was determined via Qubit4.0 (Thermo Fisher Scientific, Waltham, MA, USA, Cat. No. Q33226). DNA integrity was assessed by 1% agarose gel electrophoresis. Library construction and sequencing was carried out using an Illumina NovaSeq 6000 (Illumina Inc., San Diego, CA, USA). Whole-genome paired-end sequencing (PE150, 150 bp for each read) was conducted, with a sequencing data volume of 1 gigabase (Gb) per isolate. All gene screening cut-off values and bioinformatics software were run under the default settings without manual adjustment. The whole genome DNA was randomly fragmented to an average size of 200–400 bp. The selected fragments were through end-repair, 3’ adenylated, adapters-ligation, PCR amplifying. After purification using magnetic beads, libraries were quantified with a Qubit 4.0 fluorometer, and fragment size was evaluated via 2% agarose gel electrophoresis. After sequencing, raw reads were filtered via Trimmomatic (v0.36) (Bolger et al., 2014) by removing adaptors and low-quality reads, and clean reads were then obtained. Genome assembly was done using SPAdes (v3.15) (Bankevich et al., 2012) and gaps were filled using Gapfiller (v1.11) (Boetzer and Pirovano, 2012). Gene predictions and annotations were generated using Prokka (version 1.10) (Seemann, 2014) and the National Center for Biotechnology Information nr database. The Virulence Factor Database (http://www.mgc.ac.cn/VFs/main.htm) and Comprehensive Antibiotic Resistance Database (https://card.mcmaster.ca/) were queried to predict the virulence genes and antibiotic-resistance genes. The multilocus sequence type (ST) was identified using BIGSdb (https://bigsdb.pasteur.fr/klebsiella/) and the capsular serotype was determined using Pathogenwatch (http://pathogen.watch/). Plasmid replicon types were identified using PlasmidFinder (Carattoli et al., 2014). We reconstructed a phylogenetic tree based on core genome single-nucleotide polymorphisms (cgSNPs).

Genomic variants were identified using Snippy (v4.6.0) against the reference genome of *Klebsiella pneumoniae* strain K109 (SRA accession: SRR39530536). Only biallelic SNPs were retained, and indels were excluded. Variants detected in fewer than 20% of isolates were filtered out, while recombinant regions were not removed. Finally, 13,241 high-quality cgSNPs located in core genomic regions were obtained. We then calculated pairwise genetic distances based on these SNPs to classify isolates into different genetic clusters. Strains with pairwise core-genome SNP differences of no more than 10 SNPs were classified into the same epidemic outbreak clone; isolates with pairwise differences ranging from 25 to 30 SNPs were considered to belong to an identical clonal complex, while those differing by more than 50 SNPs were assigned to distinct phylogenetic branches.

### Statistical analysis

Statistically significant differences in resistance rates between CR-cKp and CR-hvKp strains were calculated using Pearson’s *χ²* test or Fisher’s exact test in GraphPad Prism 8. Fisher’s exact test was adopted for comparison if the expected value of any cell in the contingency table was less than 5. *P* < 0.05 was considered statistically significant.

## Results

### Clinical characteristics of CRKP in patients with BSI

A total of 100 non-repetitive CRKP isolates were collected from patients in 15 hospitals across eight cities in Hebei Province (Shijiazhuang, Baoding, Tangshan, Chengde, Handan, Zhangjiakou, Xingtai and Cangzhou) from January 2022 to December 2024 (only one non-repetitive isolate per patient was enrolled to eliminate sampling bias). All isolates were derived from blood culture specimens. The average patient age was 61 years, 61% of patients were male, and except for 17 outpatients (17%), the remaining 83 cases were inpatients (83%), with 34% of patients from the intensive care unit. Patient details are shown in **Table 2**.

**Table 2:** Basic characteristics of patients from whom CRKP strains were obtained.

Essential feature	Number of cases (n = 100)	Percentage (%)
Gender		
male	61	61
female	39	39
Category		
outpatient	17	17
inpatient	83	83
Department Distribution		
Department of Critical Care Medicine	34	34
Department of Respiratory Medicine	17	17
Department of General Practice	11	11
Department of Neurosurgery	4	4
Department of Hematology	3	3
Department of Infectious Diseases	2	2
Department of Oncology	2	2
Department of Pediatrics	2	2
Department of Geriatrics	2	2
Department of Gynecology	1	1
Department of Neurology	1	1
Department of Urology	1	1
Department of General Surgery	1	1
Department of Neonatology	1	1
Department of International Medicine	1	1

### Analysis of antimicrobial susceptibility

Nearly all CRKP strains showed resistance to commonly used antibiotics, including penicillins, cephalosporins, cephamycins, monobactams, carbapenems, quinolones, and β-lactam inhibitor combinations (resistance rate > 85%), whereas the resistance rate to aminoglycosides was lower (< 85%). The susceptibility rate to polymyxin B was 90%. Notably, the resistance rate to amikacin was 66%, and 50% of the strains remained susceptible to trimethoprim/sulfamethoxazole. The MIC results of all antimicrobial agents are presented in **Supplementary Table 1**. Notably, among all tested antimicrobial agents, CR-hvKp isolates exhibited significantly higher resistance rates to amikacin and gentamicin relative to CR-cKp isolates (*P* < 0.05). No statistically significant intergroup differences were detected for the remaining tested antibiotics (Details are shown in **Table 3**). A total of 100 CRKP strains were positive for mCIM, and 9 CRKP strains were positive for eCIM.

**Table 3:** Antimicrobial susceptibility results of 100 CRKP strains.

	CRKP (n=100)				CR-cKp (n=38)				CR-hvKp (n=62)				*P*
Antibiotics	S%	I%	R%	SDD%	S%	I%	R%	SDD%	S%	I%	R%	SDD%	
Ampicillin	0	0	100	-	0	0	100	-	0	0	100	-	> 0.999
Ampicillin/Sulbactam	0	0	100	-	0	0	100	-	0	0	100	-	> 0.999
Piperacillin	0	-	99	1	0	-	98	2	0	-	100	0	0.380
Piperacillin/Tazobactam	2	-	98	0	5	-	95	0	0	-	100	0	0.140
Cefazolin	0	0	100	-	0	0	100	-	0	0	100	-	> 0.999
Cefuroxime	0	1	99	-	0	2	98	-	0	0	100	-	0.380
Cefotetan	4	7	89	-	2	16	82	-	5	2	93	-	> 0.999
Ceftazidime	0	0	100	-	0	0	100	-	0	0	100	-	> 0.999
Ceftriaxone	0	0	100	-	0	0	100	-	0	0	100	-	> 0.999
Cefepime	0	-	100	0	0	-	100	0	0	-	100	0	> 0.999
Aztreonam	5	0	95	-	11	0	89	-	2	0	98	-	0.070
Imipenem	0	0	100	-	0	0	100	-	0	0	100	-	> 0.999
Meropenem	0	0	100	-	0	0	100	-	0	0	100	-	> 0.999
Amikacin	31	3	66	-	53	8	39	-	18	0	82	-	0.001
Gentamicin	23	3	74	-	34	8	58	-	16	0	84	-	0.026
Tobramycin	16	0	84	-	24	0	76	-	11	0	89	-	0.158
Ciprofloxacin	5	1	94	-	11	0	89	-	3	0	97	-	0.359
Levofloxacin	5	6	89	-	8	13	79	-	3	2	95	-	0.340
Trimethoprim/ tulfamethoxazole	50	0	50	-	61	0	39	-	44	0	56	-	0.596
Polymyxin B	90	0	10	-	87	0	13	-	92	0	8	-	0.410

S, susceptible; R, resistant; SDD, dose-dependent; -, not applicable; *P* values were calculated using Pearson’s *χ²* test or Fisher’s exact test to compare the differences in antimicrobial resistance rates between CR-cKp and CR-hvKp isolates. *P* < 0.05 was defined as statistically significant.

### CRKP mainly carried the *blaKPC* resistance gene

**Figure 1:**
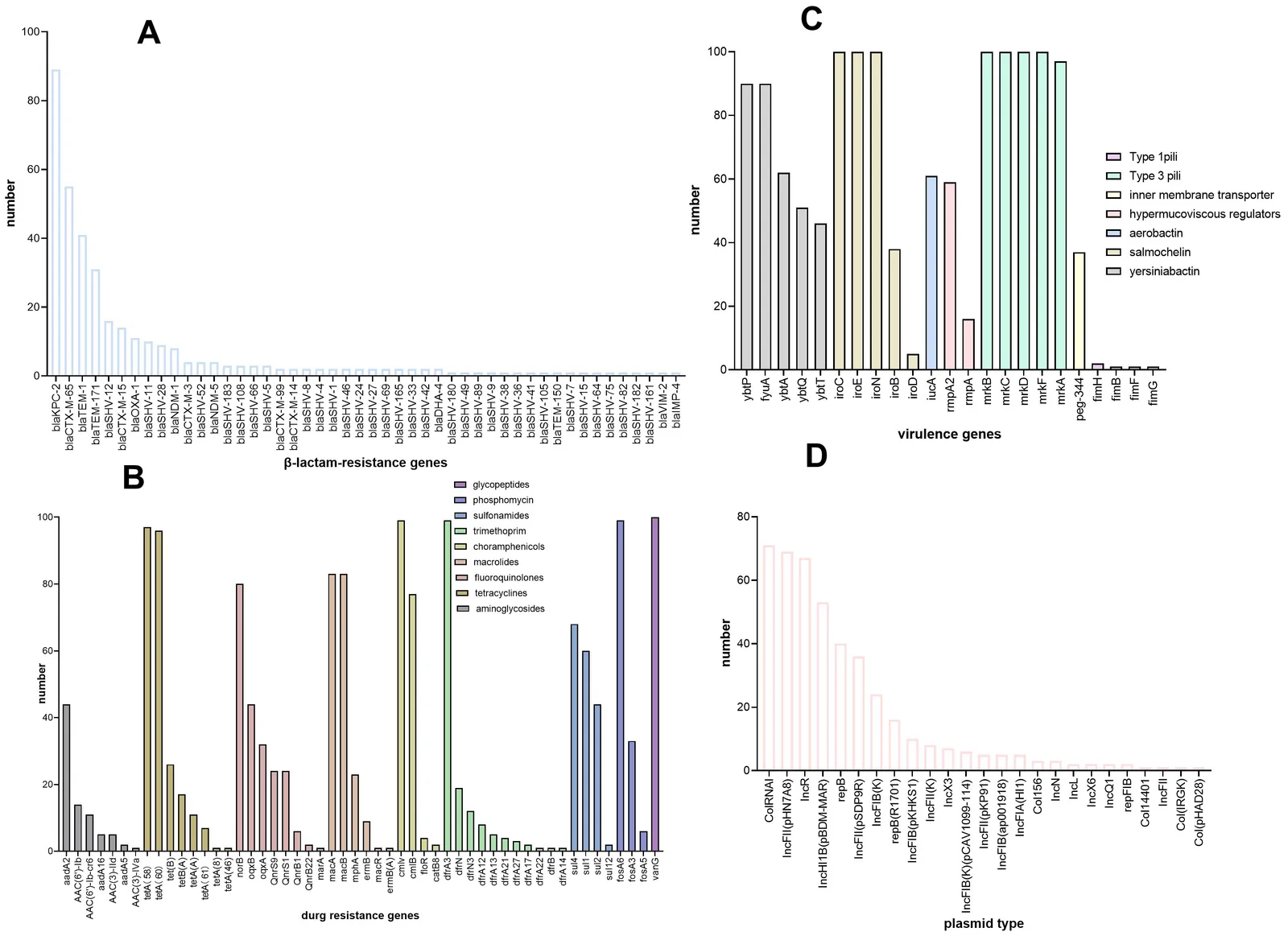
Genetic characteristics of the 100 CRKP isolates. Categories of **(A)** β-lactam resistance genes, **(B)** drug resistance genes, **(C)** virulence genes, and **(D)** plasmid replicon types harbored by these isolates.

We elucidated the antimicrobial resistance mechanisms of these isolates via PCR and WGS. All the CRKP isolates harbored carbapenemase-encoding genes, with the *blaKPC* gene being the most prevalent (89%, 89/100), followed by *blaNDM* (12%, 12/100) and *blaOXA* (11%, 11/100). In contrast, the carriage rates of *blaVIM* and *blaIMP* were the lowest (1% each, 1/100). Additionally, 3% (3/100) of the isolates co-harbored both *blaKPC* and *blaNDM*, while 8% (8/100) co-harbored *blaKPC* and *blaOXA*. Furthermore, WGS analysis identified a total of 100 resistance genes classified into 10 distinct categories among the 100 CRKP isolates (**Figures 1A, B**). Notably, all isolates carried resistance genes against β-lactams, tetracyclines, macrolides, fluoroquinolones, chloramphenicols, trimethoprim, sulfonamides, and fosfomycin (**Figures 1A, B**). Among these, β-lactam resistance genes exhibited the highest diversity (**Figure 1A**), with a total of 48 different types identified. In contrast, only 58% of the CRKP isolates harbored aminoglycoside-resistance genes.

### Presence of virulence-related genes in CRKP isolates and positive string test results

Virulence genes were further analyzed to characterize the pathogenic profiles of the isolates. In summary, a total of 90 isolates carried yersiniabactin-encoding genes (*ybt* and/or *fyu*), 61 isolates harbored the aerobactin-encoding gene (*iuc*), and all 100 isolates carried salmochelin-encoding genes (*iro*). Additionally, 62 isolates were positive for hypermucoviscosity-associated regulatory genes (*rmpA* and/or *rmpA2*). Other key virulence factors identified included type I fimbriae (*fim*), type III fimbriae (*mrk*), and the *peg-344* genes. Among the 100 CRKP isolates, the detection rates of *fim*, *mrk*, and *peg-344* were 3% (3/100), 100% (100/100), and 37% (37/100), respectively (**Figure 1C**). Based on the above criteria, 62 isolates (62%) were classified as CR-hvKp, while the remaining 38 isolates (38%) were designated as CR-cKp. Furthermore, only 13 out of 100 isolates (13%) had positive string tests, as a phenotypic indicator of hypermucoviscosity, and five of these positive isolates were confirmed as CR-hvKp strains.

### Distribution of plasmid replicon types in CRKP

PlasmidFinder analysis (Carattoli et al., 2014) detected 25 distinct plasmid replicon types among the 100 CRKP isolates (**Figure 1D**). Each isolate harbored between one and five distinct plasmid replicon types, with IncFII(pHN7A8) (69%, 69/100 isolates) and ColRNAI (71%, 71/100 isolates) being the predominant types.

### Multilocus sequence typing and capsular serotyping analysis

**Figure 2:**
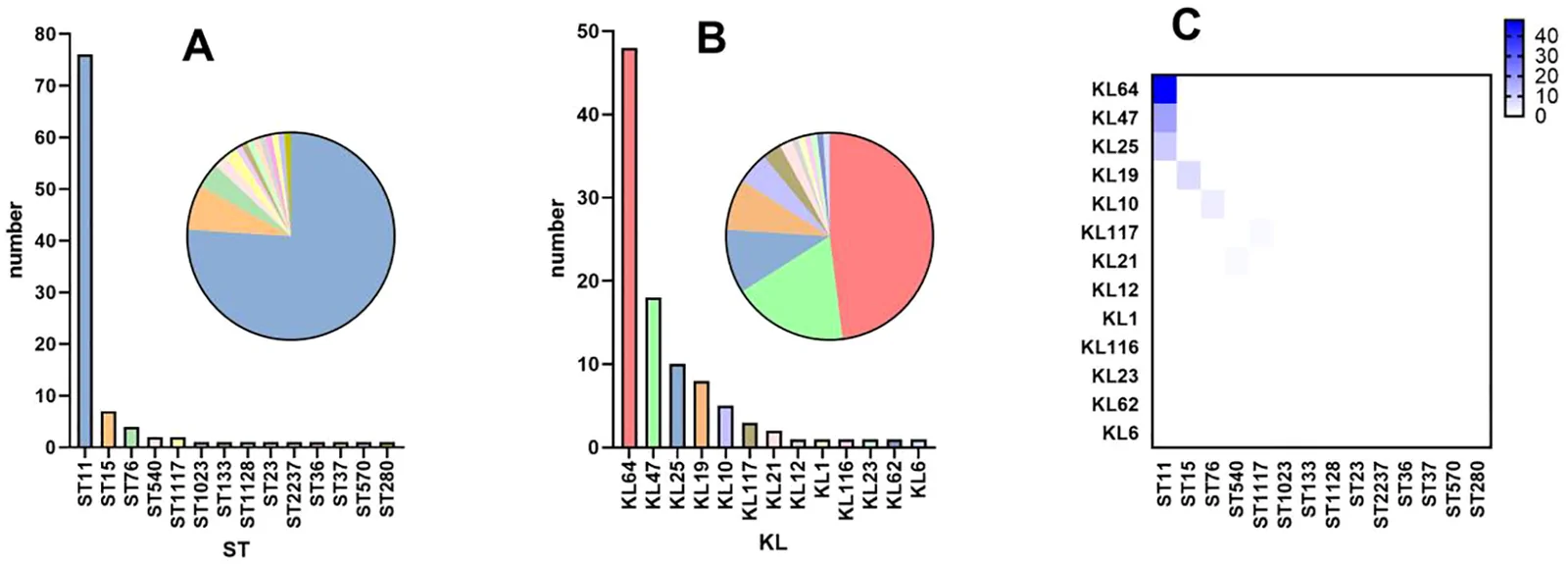
Distribution of all CRKP strains of ST and capsular serotypes. **(A)** Sequence types and **(B)** capsular serotypes of different strains. Corresponding proportions and colors in column charts and pie charts. **(C)** Association heatmap showing corresponding relationship between different serovars of ST and capsular serotypes of KL, and number of strains.

The ST and capsular serotype were analyzed using the assembled genomes. A total of 14 distinct sequence types were identified, including ST11 (76 strains, 76%), ST15 (7 strains, 7%), ST76 (4 strains, 4%), ST1128 (2 strains, 2%), ST540 (2 strains, 2%), ST1023 (1 strain, 1%), ST133 (1 strain, 1%), and other types (**Figure 2A**). Concurrently, 13 different capsular serotypes were detected, with KL64 (48 strains, 48%) and KL47 (18 strains, 18%) being the most prevalent, followed by KL25 (10 strains, 10%), KL10 (5 strains, 5%), KL19 (8 strains, 8%), and other serotypes (**Figure 2B**). Further analysis revealed that ST11-KL64 CRKP strains accounted for the highest proportion (48%, 48/100) (**Figure 2C**).

**Figure 3:**
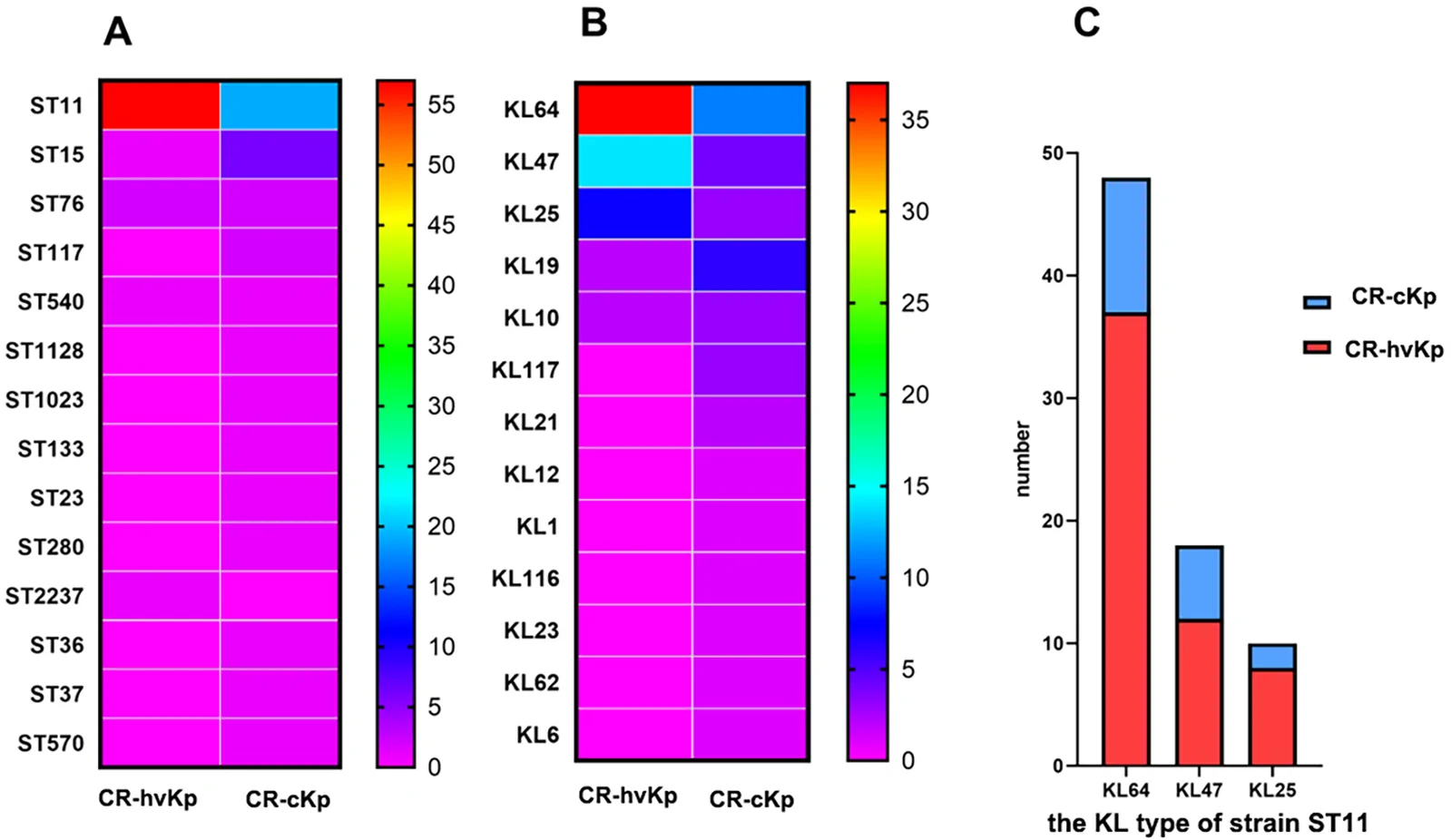
Comparison of molecular typing features between CR-hvKp and CR-cKp isolates. **(A)** Heatmap showing distribution of sequence types (STs) in CR-hvKp and CR-cKp groups. Color gradient (from purple to red) indicates number of isolates, with ST11 being the most prevalent type in both groups and showing the highest abundance in CR-hvKp. **(B)** Heatmap illustrating distribution of capsular serotypes (KL types) in CR-hvKp and CR-cKp groups. KL64 and KL47 were the dominant serotypes, with KL64 showing the highest prevalence in CR-hvKp, followed by KL47. **(C)** Stacked bar chart depicting distribution of KL types specifically among ST11 isolates, stratified by CR-hvKp (red) and CR-cKp (blue). KL64 was the most abundant serotype in ST11 isolates, with CR-hvKp accounting for the majority, followed by KL47 and KL25.

**Figure 4:**
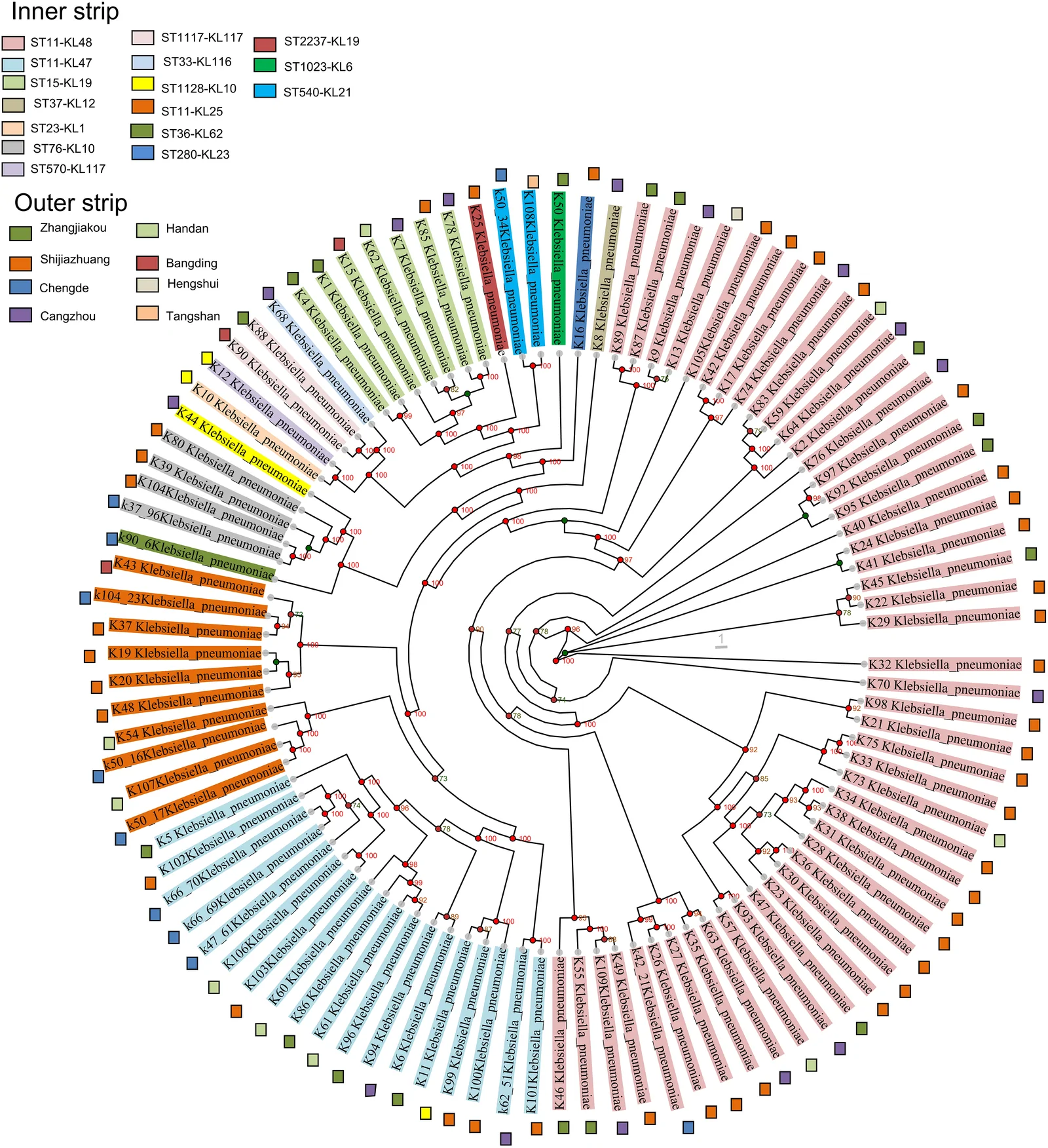
Circular core-genome SNP phylogenetic tree of carbapenem-resistant *Klebsiella pneumoniae* isolates collected from multiple cities across Hebei Province. Two layers of color strips are arranged on the periphery of the tree: the inner strips correspond to combined ST-KL capsular typing of each strain, and the outer strips represent the sampling cities within Hebei Province as annotated in the legend on the upper left.

The dominant sequence type among CR-hvKp isolates was ST11 (57 strains), followed by ST76 (2 strains), ST15 (1 strain), ST2237 (1 strain), and ST540 (1 strain). In contrast, CR-cKp isolates exhibited more-diverse sequence types, with ST11 (19 strains) as the predominant type. The subsequent prevalent sequence types were ST15 (6 strains), ST1128 (2 strains), ST76 (2 strains), ST117 (2 strains), ST1023 (1 strain), ST133 (1 strain), and other types (**Figure 3A**). The predominant serotype in all the CR-hvKp samples was KL64, followed by KL47. Notably, serotypes KL23, KL21, KL62, KL12, KL117, KL1, KL116, and KL6 were only detected in the CR-cKp isolates (**Figure 3B**). Further analysis of the capsular serotypes of 76 ST11 CRKP isolates identified three capsular serotypes, namely KL64, KL47, and KL25. It is noteworthy that KL64 was the dominant type (37 isolates) among the CR-hvKp isolates, followed by KL47 (12 isolates) (**Figure 3C**). According to the core-genome SNP analysis, the clustered large clade composed of ST11-KL64 was the predominant lineage among the sampled isolates (**Figure 4**).

## Discussion

The prevalence and spread of CRKP in BSIs pose a serious threat in terms of the safety of clinical diagnosis and treatment. The epidemiological characteristics of CRKP strains vary among regions (Wang et al., 2024), and local research data on this issue are scarce. This study conducted a systematic analysis of 100 CRKP strains from BSIs in Hebei Province, to reveal their molecular epidemiological features, clarify the local CRKP epidemic pattern and its characteristics related to drug resistance and virulence, and provide an important scientific basis for formulating localized strategies for infection prevention, control, and treatment in clinical practice.

CRKP-related BSIs are often associated with high mortality, prolonged hospital stays, and treatment failure (Zhang et al., 2018). In contrast to CRKP isolates recovered from various clinical specimens (Zhu et al., 2022), CRKP strains originating from BSIs characteristically exhibit extensive MDR (Zhong et al., 2025). In this study, most CRKP strains from BSIs were resistant to penicillins, cephalosporins, cephamycins, monobactams, carbapenems, quinolones, and β-lactam/β-lactamase inhibitor combinations. CR-hvKp developed resistance to multiple drugs, increasing the difficulty of treatment in this region.

The current results revealed the presence of 10 classes and 100 distinct resistance genes among CRKP isolates in this region. Using combined phenotypic antimicrobial susceptibility testing and genotypic resistance detection, we confirmed that carbapenemase production represented the primary resistance mechanism of CRKP in this region. In a study focusing on carbapenem-resistant *Klebsiella pneumoniae* subclones circulating in China, a total of 6,609 Carbapenemase-Resistant Enterobacterales (CRE) isolates were screened for carbapenemase genes, and over 80% of CRKP isolates were found to harbor the *blaKPC-2* gene (Wang et al., 2024). Consistent with this nationwide prevalence data, carbapenemase profiling performed in the present study confirmed that *blaKPC-2* was the predominant carbapenemase gene among local CRKP isolates. CRKP isolates carrying *blaKPC* exhibit pathogenic potential capable of causing human infections (Yang et al., 2021). The *blaKPC* gene is typically plasmid-borne and associated with diverse plasmid incompatibility (Inc) groups (Yang et al., 2021). In addition, only one isolate harboring the carbapenem-resistance gene *blaIMP-4* was identified. Previous evidence indicates that *blaIMP-4* can be located on independent plasmids, self-conjugative fusion plasmids, or the bacterial chromosome (Shi et al., 2024). Specifically, *blaIMP-4* on fusion plasmids and the chromosome is generated by intact plasmid recombination mediated by IS26 and ltrA, respectively, and the genomic position of *blaIMP-4* did not affect its stability (Shi et al., 2024). Although the present study was unable to determine definitively has not been determined whether it is located on the plasmid, our findings provide new insights to support future investigations in the region. Notably, we detected three CRKP isolates coharboring *blaKPC* and *blaNDM* (designated KN-CRKP). Previous studies have demonstrated that CRKP isolates coharboring *blaKPC-2* and *blaNDM-1* (K2N1-CRKP), *blaKPC-2* and *blaNDM-5* (K2N5-CRKP) and *blaKPC-3* and *blaNDM-1* (K3N1-CRKP) display distinct geographic distribution patterns: K2N1-CRKP and K2N5-CRKP mainly circulate in China, whereas K3N1-CRKP predominates in the United States (Zhang et al., 2025). In our present work, three KN-type CRKP isolates were recovered, among which two were identified as K2N1-CRKP and the remaining one as K2N5-CRKP.

Historically, the hypermucoviscous phenotype has been regarded as the hallmark characteristic of hvKp (Choby et al., 2020), with the string test commonly used as a rapid phenotypic screening tool. However, as not all hvKp isolates exhibit the hypermucoviscous phenotype, and this trait can also be observed in some cKp strains (Catalán-Nájera et al., 2017; Russo et al., 2018). Consistent with these findings, our data showed that only 5 out of 13 hypermucoviscous CRKP isolates were confirmed as hvKp strains. This discrepancy highlights the limitations of relying solely on the string test for hvKp identification. Such misclassification not only introduces confusion into the published literature but also hinders the accurate assessment of CR-hvKp epidemiology and the development of targeted prevention strategies. Increasing reports have described CR-hvKp isolates with both high-level drug resistance and hypervirulence (Yang et al., 2022; Zhao et al., 2023). Such superbugs not only cause antibiotic-refractory infections, but also aggravate disease severity (Zhao et al., 2023). According to the screening criteria defined by Lam et al. (2021), a high detection rate of CR-hvKp was observed in Hebei region, with a total of 62 CR-hvKp isolates (62%) identified in our study. Consistently, a previous investigation of 320 CRKP clinical isolates reported 179 CR-hvKp strains, further indicating a widespread distribution of hypervirulent clones among carbapenem-resistant *Klebsiella pneumoniae* populations (Zhao et al., 2023). The emergence of CR-hvKp is attributed to the continuous evolution of plasmids carrying carbapenem-resistance genes or hypervirulence-associated genes (Danxia Gu, 2021). The coexistence of multiple plasmids in a single isolate is a typical genetic characteristic of CR-hvKp, enabling strains to simultaneously acquire carbapenem resistance and hypervirulence, and accelerating the epidemic spread and evolution of high-risk clones through horizontal plasmid typestransmission (Sun et al., 2025). The present results showed that 95% of the CRKP isolates (CR-cKp and CR-hvKp) carried at least two plasmid replicon types, indicating that CRKP can carry multiple plasmid replicon types, which may greatly facilitate the widespread dissemination of resistance genes and increase the risk of nosocomial outbreaks.

The prevalent STs of *K. pneumoniae* vary globally. In China, previous studies have identified ST11 as the dominant endemic clone, which is frequently associated with carbapenem resistance (Li et al., 2014; Lee et al., 2016; Wang et al., 2018), while ST258 and ST307, which are also carbapenem-resistant, have been reported as the predominant clones in the United States (Kitchel et al., 2009; Russo et al., 2018; Kochan et al., 2022; Shropshire et al., 2022). The present study revealed a high prevalence of ST11-type CRKP strains in Hebei region, and all ST11 isolates harbored the *blaKPC* resistance gene, while ST258 and ST307 were not detected. According to the core-genome SNP analysis, the clustered large clade composed of ST11-KL64 was the predominant lineage among the sampled isolates. Consistent with the national trend (Hu et al., 2024), KL64 was the most prevalent serotype in Hebei Province, China, followed by KL47. Notably, KL64 isolates carry more resistance and virulence determinants (Zhao et al., 2020), and the iron carrier production of the KL64 type CR-hvKp isolate was significantly higher than that of the KL47 type isolate (Zhao et al., 2023). High-level siderophores can enhance a bacterium’s ability to acquire iron in the host’s blood, promoting their survival and proliferation (Fang et al., 2025). In the present study, KL64 was identified as the dominant capsular serotype among CRKP isolates derived from bloodstream infection patients in Hebei Province. Previous studies have demonstrated that compared with patients with KL47-related BSIs, patients with KL64-related BSIs had a higher risk of sepsis (Zhou et al., 2021). Accordingly, intensified clinical surveillance and optimized treatment strategies for KL64 CRKP infections might be considered, in light of previous clinical evidence. As previously reported (Shi, 2024), the dominant clone ST11 has undergone subclone replacement in Chinese clinical settings in recent years, with ST11-KL47 being gradually replaced by ST11-KL64. According to core-genome SNP analysis, the current study identified that ST11-KL64 was the predominant lineage among the sampled isolates. Meanwhile, most CR-hvKp isolates belonged to the ST11-KL64 lineage, indicating that ST11-KL64 may be one of the major clonal lineages driving the local spread of CR-hvKp in this region. Its ongoing dissemination poses potential threats to clinical management and public health.

Several limitations of this study should be noted. First, the relatively small sample size limits the generalizability of the findings. Second, owing to the strict information confidentiality regulations of the participating hospital, we are not allowed to publish the following data in the manuscript: the total number of *Klebsiella pneumoniae* bloodstream isolates during the study period, the proportion of CRKP among all *Klebsiella pneumoniae* BSI isolates, as well as detailed isolate counts stratified by hospital and year. In addition, our study has certain methodological limitations. Since the genomic analysis was performed using short-read sequencing data, genetic characterization depended on sequence similarity alignment and *de novo* assembly. Consequently, we were unable to identify the precise physical positions of the target genes on the bacterial genome. Furthermore, We were unable to distinguish specific plasmid categories (i.e., virulence plasmids and resistance plasmids), nor could we confirm whether the target genes were plasmid-borne.

This study systematically characterized the epidemiological and molecular features of CRKP responsible for BSIs in Hebei Province, China, and identified ST11-KL64 was the predominant lineage among the sampled isolates. This clone harbors a large repertoire of MDR and virulence genes, with a high prevalence of CR-hvKp, which poses challenges to clinical anti-infective therapy. Our findings supplement the regional epidemiological data on CRKP isolates from BSIs in Hebei, providing targeted evidence for local medical institutions to optimize infection prevention and control strategies and antibiotic stewardship programs. Furthermore, this study establishes a molecular and epidemiological foundation for further investigations into the transmission mechanisms and evolutionary dynamics of the ST11-KL64 clone, and offers public health insights for reducing the morbidity and mortality of CRKP infections in northern China.

## Data Availability

The datasets presented in this study can be found in online repositories. The names of the repository/repositories and accession number(s) can be found in the article/**Supplementary Material**.
