# Supplementary material for: Regional surveillance of carbapenem-resistant Klebsiella pneumoniae responsible for bloodstream infections in Hebei province
Source: Front Cell Infect Microbiol. 2026 Aug 14;16:1883044. doi: 10.3389/fcimb.2026.1883044 (PMC13521795; doi:10.3389/fcimb.2026.1883044)
Supplement: Supplementary file 1 [file DataSheet1.pdf]

Supplementary Table 1. The MIC results of all antimicrobial agents

number	Ampicillin	Ampicillin/Sulbactam	Piperacillin	Piperacillin/Tazobactam	Cefazolin	Cefuroxime	Cefotetan	Ceftazidime	Ceftriaxone	Cefepime	Aztreonam	Imipenem	Meropenem	Amikacin	Gentamicin	Tobramycin	Ciprofloxacin	Levofloxacin	Trimethoprim / tulfamethoxazole	Polymyxin B
k1	≥ 32	≥ 32	≥ 128	64	≥ 64	≥ 64	32	≥ 64	≥ 64	≥ 64	≥ 64	≥ 16	≥ 16	16	≥ 16	≥ 16	≥ 4	4	≤ 20	0.25
K2	≥ 32	≥ 32	≥ 128	≥ 128	≥ 64	≥ 64	≥ 64	≥ 64	≥ 64	≥ 64	≥ 64	≥ 16	≥ 16	4	≥ 16	≥ 16	≥ 4	4	≤ 20	1
K4	≥ 32	≥ 32	> 128	≥ 128	≥ 64	≥ 64	≥ 64	≥ 64	≥ 64	≥ 64	≥ 64	≥ 16	≥ 16	4	≥ 16	≥ 16	≥ 4	4	≤ 20	0.125
K5	≥ 32	≥ 32	≥ 128	≥ 128	≥ 64	≥ 64	≥ 64	≥ 64	≥ 64	≥ 64	≥ 64	≥ 16	≥ 16	≥ 64	≥ 16	≥ 16	≥ 4	≥ 8	≤ 20	0.03125
K6	≥ 32	≥ 32	≥ 128	≥ 128	≥ 64	≥ 64	≥ 64	≥ 64	≥ 64	≥ 64	≥ 64	≥ 16	≥ 16	≥ 64	≥ 16	≥ 16	≥ 4	≥ 8	≤ 20	> 16
K7	≥ 32	≥ 32	≥ 128	≥ 128	≥ 64	≥ 64	≥ 64	≥ 64	≥ 64	≥ 64	≥ 64	≥ 64	≥ 16	≥ 16	8	≥ 16	≥ 16	≥ 4	≤ 20	0.5
K8	≥ 32	≥ 32	≥ 128	≥ 128	≥ 64	≥ 64	32	≥ 64	≥ 64	8	4	≥ 16	8	≤ 2	≤ 1	≤ 1	1	1	≤ 20	0.0625
K9	≥ 32	≥ 32	≥ 128	64	≥ 64	≥ 64	32	≥ 64	≥ 64	≥ 64	≥ 64	16	16	2	< 1	8	≥ 4	≥ 8	≤ 20	0.125
K10	≥ 32	≥ 32	≥ 128	≥ 128	≥ 64	≥ 64	32	≥ 64	≥ 64	8	≥ 64	≥ 16	8	≤ 2	≤ 1	≤ 1	≤ 0.25	≤ 0.25	≤ 20	0.125
K11	≥ 32	≥ 32	≥ 128	≥ 128	≥ 64	≥ 64	≥ 64	≥ 64	≥ 64	≥ 64	≥ 64	≥ 16	≥ 16	≥ 64	≥ 16	≥ 16	≥ 4	≥ 8	≤20	0.0625
K12	≥ 32	≥ 32	≥ 128	≥ 128	≥ 64	≥ 64	≥ 64	≥ 64	≥ 64	≥ 64	≥ 64	8	8	≤2	≥ 16	8	1	1	≥ 320	16
K13	≥ 32	≥ 32	≥ 128	≥ 128	≥ 64	≥ 64	≥ 64	≥ 64	≥ 64	16	≥ 64	≥ 16	≥ 16	≥ 64	8	≥ 16	≥ 4	≥ 8	≥ 320	> 16
K15	≥ 32	≥ 32	≥ 128	≥ 128	≥ 64	≥ 64	≥ 64	≥ 64	≥ 64	≥ 64	≥ 64	≥ 16	≥ 16	≤ 2	≥ 16	8	≥ 4	4	≤20	0.25

K16	≥32	≥32	≥128	64	≥64	≥64	≤4	≥64	≥64	32	≥64	8	8	2	≤1	8	≥4	1	≥320	0.5
K17	≥32	≥32	≥128	≥128	≥64	≥64	≥64	≥64	≥64	≥64	≥64	≥16	≥16	≥64	≥16	≥16	≥4	≥8	≥320	>16
K19	≥32	≥32	≥128	≥128	≥64	≥64	≥64	≥64	≥64	≥64	≥64	≥16	≥16	≥64	≥16	≥16	≥4	≥8	≥320	0.125
K20	≥32	≥32	≥128	≥128	≥64	≥64	≥64	≥64	≥64	≥64	≥64	≥16	≥16	≥64	≥16	≥16	≥4	≥8	≥320	0.125
K21	≥32	≥32	≥128	≥128	≥64	≥64	≥64	≥64	≥64	≥64	≥64	≥16	≥16	≤2	≤1	≤1	≥4	≥8	≤20	0.5
K22	≥32	≥32	≥128	≥128	≥64	≥64	≥64	≥64	≥64	≥64	≥64	≥16	≥16	≥64	≥16	≥16	≥4	≥8	≥320	2
K23	≥32	≥32	≥128	≥128	≥64	≥64	≥64	≥64	≥64	≥64	≥64	≥16	≥16	≥64	≥16	≥16	≥4	≥8	≥320	0.125
K24	≥32	≥32	≥128	≥128	≥64	≥64	≥64	≥64	≥64	≥64	≥64	≥16	≥16	≥64	≥16	≥16	≥4	≥8	≥320	0.125
K25	≥32	≥32	≥128	≥128	≥64	≥64	≥64	≥64	≥64	≥64	≥64	≥16	≥16	≥64	≥16	≥16	≥4	≥8	≤20	0.25
K26	≥32	≥32	≥128	≥128	≥64	≥64	≥64	≥64	≥64	≥64	≥64	≥16	≥16	≥64	≥16	≥16	≥4	≥8	≤20	0.25
K27	≥32	≥32	≥128	≥128	≥64	≥64	≥64	≥64	≥64	≥64	≥64	≥16	≥16	≥64	≥16	≥16	≥4	≥8	40	0.125
K28	≥32	≥32	≥128	≥128	≥64	≥64	≥64	≥64	≥64	≥64	≥64	≥16	≥16	≥64	≥16	≥16	≥4	≥8	≥320	0.25
K29	≥32	≥32	≥128	≥128	≥64	≥64	≥64	≥64	≥64	≥64	≥64	≥16	≥16	≥64	≥16	≥16	≥4	≥8	≥320	0.5
K30	≥32	≥32	≥128	≥128	≥64	≥64	≥64	≥64	≥64	≥64	≥64	≥16	≥16	≥64	≥16	≥16	≥4	≥8	≥320	0.25
K31	≥32	≥32	≥128	≥128	≥64	≥64	≥64	≥64	≥64	≥64	≥64	≥16	≥16	≥64	≥16	≥16	≥4	≥8	≥320	0.25
K32	≥32	≥32	≥128	≥128	≥64	≥64	≥64	≥64	≥64	≥64	≥64	≥16	≥16	≥64	≥16	≥16	≥4	≥8	≥320	0.0625

K33	≥ 32	≤ 2	≤ 4	≤4	≤ 4	≤1	≤4	≤ 1	≤ 1	≤ 1	≤ 1	≥ 16	≥ 16	≤ 2	≤ 1	≤ 1	≤ 0.25	≤ 0.25	≤ 20	0.5
K34	≥ 32	≥ 32	≥ 128	≥ 128	≥ 64	≥ 64	≥ 64	≥ 64	≥ 64	≥ 64	≥ 64	≥ 16	≥ 16	≥ 64	≥ 16	≥ 16	≥ 4	≥ 8	≥ 320	>16
K35	≥ 32	≥ 32	≥ 128	≥ 128	≥ 64	≥ 64	≥ 64	≥ 64	≥ 64	≥ 64	≥ 64	≥ 16	≥ 16	≤ 2	≤ 1	≤ 1	≥ 4	≥ 8	≥ 320	0.0078125
K36	≥ 32	≥ 32	≥ 128	≥ 128	≥ 64	≥ 64	≥ 64	≥ 64	≥ 64	≥ 64	≥ 64	≥ 16	≥ 16	≥ 64	≥ 16	≥ 16	≥ 4	≥ 8	≥ 320	0.25
K37	≥ 32	≥ 32	≥ 128	≥ 128	≥ 64	≥ 64	≥ 64	≥ 64	≥ 64	≥ 64	≥ 64	≥ 16	≥ 16	≥ 64	≥ 16	≥ 16	≥ 4	≥ 8	40	0.125
K38	≥ 32	≥ 32	≥ 128	≥ 128	≥ 64	≥ 64	≥ 64	≥ 64	≥ 64	≥ 64	≥ 64	≥ 16	≥ 16	≥ 64	≥ 16	≥ 16	≥ 4	≥ 8	≥ 320	0.125
K39	≥ 32	≥ 32	≥ 128	≥ 128	≥ 64	≥ 64	≥ 64	≥ 64	≥ 64	≥ 64	≥ 64	≥ 16	≥ 16	≤ 2	≤ 1	≤ 1	≤ 0.25	≤ 0.25	≤ 20	> 16
K40	≥ 32	≥ 32	≥ 128	≥ 128	≥ 64	≥ 64	≥ 64	≥ 64	≥ 64	≥ 64	≥ 64	≥ 16	≥ 16	≥ 64	≥ 16	≥ 16	≥ 4	≥ 8	≥ 320	1
K41	≥ 32	≥ 32	≥ 128	≥ 128	≥ 64	≥ 64	≥ 64	≥ 64	≥ 64	≥ 64	≥ 64	≥ 16	≥ 16	≥ 64	≥ 16	≥ 16	≥ 4	≥ 8	≥ 320	0.5
K42	≥ 32	≥ 32	≥ 128	≥ 128	≥ 64	≥ 64	≥ 64	≥ 64	≥ 64	≥ 64	≥ 64	≥ 16	≥ 16	≥ 64	≥ 16	≥ 16	≥ 4	≥ 8	40	0.25
K43	≥ 32	≥ 32	≥ 128	≥ 128	≥ 64	≥ 64	≥ 64	≥ 64	≥ 64	≥ 64	≥ 64	≥ 16	≥ 16	≥ 64	≥ 16	≥ 16	≥ 4	≥ 8	40	0.25
K44	≥ 32	≥ 32	≥ 128	≥ 128	≥ 64	≥ 64	≥ 64	≥ 64	≥ 64	≥ 64	16	≥ 16	≥ 16	≥ 64	≥ 16	≥ 16	≥ 4	≥ 8	≥ 320	0.125
K45	≥ 32	≥ 32	≥ 128	≥ 128	≥ 64	≥ 64	≥ 64	≥ 64	≥ 64	≥ 64	≥ 64	≥ 16	≥ 16	≥ 64	≥ 16	≥ 16	≥ 4	≥ 8	≥ 320	0.5
K46	≥ 32	≥ 32	≥ 128	≥ 128	≥ 64	≥ 64	≥ 64	≥ 64	≥ 64	≥ 64	≥ 64	≥ 16	≥ 16	≥ 64	≥ 16	≥ 16	≥ 4	≥ 8	≥ 320	0.5
K47	≥ 32	≥ 32	≥ 128	≥ 128	≥ 64	≥ 64	≥ 64	≥ 64	≥ 64	≥ 64	≥ 64	≥ 16	≥ 16	≥ 64	≥ 16	≥ 16	≥ 4	≥ 8	≥ 320	0.125
K48	≥ 32	≥ 32	≥ 128	≥ 128	≥ 64	≥ 64	≥ 64	≥ 64	≥ 64	≥ 64	≥ 64	≥ 16	≥ 16	≥ 64	≥ 16	≥ 16	≥ 4	≥ 8	≥ 320	0.125

K49	≥ 32	≥ 32	≥ 128	≥ 128	≥ 64	≥ 64	≥ 64	≥ 64	≥ 64	≥ 64	≥ 64	≥ 16	≥ 16	≥ 64	≥ 16	≥ 16	≥ 4	≥ 8	≤ 20	0.5
K50	≥ 32	≥ 32	≥ 128	≥ 128	≥ 64	≥ 64	32	≥ 64	≥ 64	≥ 64	32	≥ 16	8	≤ 2	≤1	≤ 1	≤ 0.25	≤ 0.25	≤ 20	0.5
K54	≥ 32	≥ 32	≥ 128	≥ 128	≥ 64	≥ 64	≥ 64	16	≥ 64	≥ 64	≥ 64	≥ 16	≥ 16	≥ 64	≥ 16	≥ 16	≥ 4	≥ 8	≤ 20	0.125
K55	≥ 32	≥ 32	≥ 128	≥ 128	≥ 64	≥ 64	≥ 64	≥ 64	≥ 64	≥ 64	≥ 64	≥ 16	≥ 16	≥ 64	≥ 16	≥ 16	≥ 4	≥ 8	≤ 20	> 16
K57	≥ 32	≥ 32	≥ 128	≥ 128	≥ 64	≥ 64	≥ 64	≥ 64	≥ 64	≥ 64	≥ 64	≥ 16	≥ 16	≥ 64	≥ 16	≥ 16	≥ 4	≥ 8	≥ 320	0.5
K59	≥ 32	≥ 32	≥ 128	≥ 128	≥ 64	≥ 64	≥ 64	≥ 64	≥ 64	≥ 64	≥ 64	≥ 16	≥ 16	≥ 64	≥ 16	≥ 16	≥ 4	≥ 8	≤ 20	0.25
K60	≥ 32	≥ 32	≥ 128	≥ 128	≥ 64	≥ 64	≥ 64	≥ 64	≥ 64	≥ 64	≥ 64	≥ 16	≥ 16	≥ 64	≥ 16	≥ 16	≥ 4	≥ 8	80	0.5
K61	≥ 32	≥ 32	≥ 128	≥ 128	≥ 64	≥ 64	≥ 64	≥ 64	≥ 64	≥ 64	≥ 64	≥ 16	≥ 16	≤ 2	≤ 1	≥ 16	≥ 4	≥ 8	≤ 20	0.03125
K62	≥ 32	≥ 32	32	≤ 4	8	16	≤ 4	≤ 1	≤ 1	≤ 1	≤ 1	8	16	≤ 2	≥ 16	8	≥ 4	≥ 8	≤ 20	0.03125
K63	≥ 32	≥ 32	≥ 128	≥ 128	≥ 64	≥ 64	16	≥ 64	≥ 64	≥ 64	≥ 64	≥ 16	≥ 16	≥ 64	≥ 16	≥ 16	≥ 4	≥ 8	≥ 320	1
K64	≥ 32	≥ 32	≥ 128	≥ 128	≥ 64	≥ 64	≥ 64	≥ 64	≥ 64	≥ 64	≥ 64	≥ 16	≥ 16	≥ 64	≥ 16	≥ 16	≥ 4	≥ 8	≥ 320	0.5
K68	≥ 32	≥ 32	≥ 128	≥ 128	≥ 64	≥ 64	≥ 64	≥ 64	≥ 64	≥ 64	≥ 64	8	≥ 16	8	≤1	8	≥ 4	4	≥ 320	16
K70	≥ 32	≥ 32	≥ 128	≥ 128	≥ 64	≥ 64	≥ 64	≥ 64	≥ 64	≥ 64	≥ 64	≥ 16	≥ 16	≥ 64	≥ 16	≥ 16	≥ 4	≥ 8	≥ 320	0.5
K73	≥ 32	≥ 32	≥ 128	≥ 128	≥ 64	≥ 64	≥ 64	≥ 64	≥ 64	≥ 64	≥ 64	≥ 16	≥ 16	≥ 64	≥ 16	≥ 16	≥ 4	≥ 8	≥ 320	0.5
K74	≥ 32	≥ 32	≥ 128	≥ 128	≥ 64	≥ 64	≥ 64	≥ 64	≥ 64	≥ 64	≥ 64	≥ 16	≥ 16	≥ 64	≥ 16	≥ 16	≥ 4	≥ 8	≥ 320	2
K75	≥ 32	≥ 32	≥ 128	≥ 128	≥ 64	≥ 64	≥ 64	≥ 64	≥ 64	≥ 64	≥ 64	≥ 16	≥ 16	≤2	≤1	≤1	≥ 4	≥ 8	≥ 320	0.5

K76	≥32	≥32	≥128	≥128	≥64	≥64	≥64	≥64	≥64	≥64	≥64	≥16	≥16	≥64	≥16	≥16	≥4	≥8	≤20	0.25
K78	≥32	≥32	≥128	≥128	≥64	≥64	≥64	≥64	≥64	≥64	≥64	≥16	≥16	≤2	≥16	8	≥4	≥8	≤20	0.125
K80	≥32	≥32	16	8	≥64	≥64	≥64	≥64	≥64	≥64	≤1	≥16	≥16	≤2	≤1	≤1	1	1	≤20	0.125
K83	≥32	≥32	≥128	≥128	≥64	≥64	≥64	≥64	≥64	≥64	≥64	≥16	≥16	≥64	≥16	≥16	≥4	≥8	≥320	1
K85	≥32	≥32	≥128	≥128	≥64	≥64	≥64	≥64	≥64	≥64	≥64	≥16	≥16	≤2	≥16	8	≥4	≥8	≤20	0.5
K86	≥32	≥32	≥128	≥128	≥64	≥64	≥64	≥64	≥64	≥64	≥64	≥16	≥16	8	≥16	≥16	≥4	≥8	≥320	0.125
K87	≥32	≥32	≥128	≥128	≥64	≥64	≥64	≥64	≥64	≥64	≥64	≥16	≥16	≤2	≤1	8	≥4	≥8	≤20	0.0625
K88	≥32	≥32	≥128	≥128	≥64	≥64	≥64	≥64	≥64	16	≥64	≥16	8	≤2	4	8	≥4	≥8	≥320	0.5
K89	≥32	≥32	≥128	≥128	≥64	≥64	≥64	≥64	≥64	≥64	≥64	≥16	≥16	≤2	≤1	8	≥4	≥8	≤20	0.0625
K90	≥32	≥32	≥128	≥128	≥64	≥64	≥64	≥64	≥64	16	≥64	≥16	8	≤2	4	8	≥4	≥8	≥320	0.5
K92	≥32	≥32	≥128	≥128	≥64	≥64	≥64	≥64	≥64	16	≥64	≥16	8	≤2	4	8	≥4	≥8	≥320	0.5
K93	≥32	≥32	≥128	≥128	≥64	≥64	≥64	≥64	≥64	≥64	≥64	≥16	≥16	≥64	≥16	≥16	≥4	≥8	≥320	>16
K94	≥32	≥32	≥128	≥128	≥64	≥64	≥64	≥64	16	16	≥64	≥16	≥16	≥64	≥16	≥16	≥4	≥8	≤20	0.25
K95	≥32	≥32	≥128	≥128	≥64	≥64	≥64	≥64	≥64	≥64	≥64	≥16	≥16	≥64	8	≥16	≥4	≥8	≥320	0.25
K96	≥32	≥32	≥128	≥128	≥64	≥64	≥64	≥64	≥64	≥64	≥64	≥16	≥16	≤2	≤1	≥16	≥4	≥8	≤20	0.125
K97	≥32	≥32	≥128	≥128	≥64	≥64	≥64	≥64	≥64	≥64	≥64	≥16	≥16	≤2	≤1	≤1	≥4	≥8	≥320	16

K98	≥ 32	≥ 32	≥ 128	≥ 128	≥ 64	≥ 64	≥ 64	≥ 64	≥ 64	≥ 64	≥ 64	≥ 16	≥ 16	≤ 2	≤ 1	≤ 1	≥ 4	≥ 8	≥ 320	1
K99	≥ 32	≥ 32	≥ 128	≥ 128	≥ 64	≥ 64	≥ 64	≥ 64	≥ 64	≥ 64	≥ 64	≥ 16	≥ 16	≥ 64	≥ 16	≥ 16	≥ 4	≥ 8	≤ 20	0.25
K100	≥ 32	≥ 32	≥ 128	≥ 128	≥ 64	≥ 64	≥ 64	≥ 64	≥ 64	≥ 64	≥ 64	≥ 16	≥ 16	≥ 64	≥ 16	≥ 16	≥ 4	≥ 8	≤ 20	0.5
K101	≥ 32	≥ 32	≥ 128	≥ 128	≥ 64	≥ 64	≥ 64	≥ 64	≥ 64	≥ 64	≥ 64	≥ 16	≥ 16	≥ 64	≥ 16	≥ 16	≥ 4	≥ 8	≤ 20	0.125
K102	≥ 32	≥ 32	≥ 128	≥ 128	≥ 64	≥ 64	≥ 64	≥ 64	≥ 64	≥ 64	≥ 64	≥ 16	≥ 16	≥ 64	≥ 16	≥ 16	≥ 4	≥ 8	≤ 20	0.125
K103	≥ 32	≥ 32	≥ 128	≥ 128	≥ 64	≥ 64	≤ 4	≥ 64	≥ 64	≥ 64	≥ 64	≥ 16	≥ 16	≥ 64	≥ 16	≥ 16	≥ 4	≥ 8	≤ 20	0.125
K104	≥ 32	≥ 32	≥ 128	≥ 128	≥ 64	≥ 64	≥ 64	≥ 64	≥ 64	≥ 64	≥ 64	≥ 16	≥ 16	≤ 2	≤ 1	≤ 1	≤ 0.25	≤ 0.25	≥ 320	0.125
K105	≥ 32	≥ 32	≥ 128	≥ 128	≥ 64	≥ 64	≥ 64	≥ 64	≥ 64	≥ 64	≥ 64	≥ 16	≥ 16	≥ 64	≥ 16	≥ 16	≥ 4	≥ 8	≥ 320	0.125
K106	≥ 32	≥ 32	≥ 128	≥ 128	≥ 64	≥ 64	≥ 64	≥ 64	≥ 64	≥ 64	≥ 64	≥ 16	≥ 16	≥ 64	≥ 16	≥ 16	≥ 4	≥ 8	≤ 20	0.125
K107	≥ 32	≥ 32	≥ 128	≥ 128	≥ 64	≥ 64	≥ 64	≥ 64	≥ 64	≥ 64	≥ 64	≥ 16	≥ 16	≥ 64	≥ 16	≥ 16	≥ 4	≥ 8	≤ 20	0.125
K108	≥ 32	≥ 32	≥ 128	64	≥ 64	≥ 64	32	≥ 64	≥ 64	≥ 64	2	≥ 16	8	≤ 2	≤ 1	2	1	1	≥ 320	0.25
K109	≥ 32	≥ 32	≥ 128	≥ 128	≥ 64	≥ 64	≥ 64	≥ 64	≥ 64	≥ 64	≥ 64	≥ 16	≥ 16	≤ 2	≤ 1	≤ 1	≥ 4	≥ 8	≤ 20	0.25
K62-51	≥ 32	≥ 32	≥ 128	≥ 128	≥ 64	≥ 64	≥ 64	≥ 64	≥ 64	≥ 64	≥ 64	≥ 16	≥ 16	≥ 64	≥ 16	≥ 16	≥ 4	≥ 8	≤ 20	0.5
K66-69	≥ 32	≥ 32	≥ 128	≥ 128	≥ 64	≥ 64	≥ 64	≥ 64	≥ 64	≥ 64	≥ 64	≥ 16	≥ 16	≥ 64	≥ 16	≥ 16	≥ 4	≥ 8	≤ 20	0.25
K66-70	≥ 32	≥ 32	≥ 128	≥ 128	≥ 64	≥ 64	≥ 64	≥ 64	≥ 64	≥ 64	≥ 64	≥ 16	≥ 16	≥ 64	≥ 16	≥ 16	≥ 4	≥ 8	≤ 20	0.5

K42-21	≥ 32	≥ 32	≥ 128	≥ 128	≥ 64	≥ 64	≥ 64	≥ 64	≥ 64	≥ 64	≥ 64	≥ 16	≥ 16	≥ 64	≥ 16	≥ 16	≥ 4	≥ 8	≥ 320	0.25
K37-96	≥ 32	≥ 32	≥ 128	≥ 128	≥ 64	≥ 64	≥ 64	≥ 64	≥ 64	≥ 64	≥ 64	≥ 16	≥ 16	≤ 2	≤ 1	≤ 1	≤ 0.25	≤ 0.25	≥ 320	0.5
K47-61	≥ 32	≥ 32	≥ 128	≥ 128	≥ 64	≥ 64	≥ 64	≥ 64	≥ 64	≥ 64	≥ 64	≥ 16	≥ 16	≥ 64	≥ 16	≥ 16	≥ 4	≥ 8	≤ 20	1
K50-16	≥ 32	≥ 32	≥ 128	≥ 128	≥ 64	≥ 64	≥ 64	≥ 64	≥ 64	≥ 64	≥ 64	≥ 16	≥ 16	≥ 64	≥ 16	≥ 16	≥ 4	≥ 8	≤ 20	0.5
K50-17	≥ 32	≥ 32	≥ 128	≥ 128	≥ 64	≥ 64	≥ 64	≥ 64	≥ 64	≥ 64	≥ 64	≥ 16	≥ 16	≥ 64	≥ 16	≥ 16	≥ 4	≥ 8	≤ 20	1
K51-34	≥ 32	≥ 32	≥ 128	64	≥ 64	≥ 64	32	≥ 64	≥ 64	8	2	≥ 16	8	≤ 2	≤ 1	2	1	1	≥ 320	0.25
K104-23	≥ 32	≥ 32	≥ 128	≥ 128	≥ 64	≥ 64	≥ 64	≥ 64	≥ 64	≥ 64	≥ 64	≥ 16	≥ 16	≥ 64	≥ 16	≥ 16	≥ 4	≥ 8	40	0.25
K90-6	≥ 32	≥ 32	≥ 128	≥ 128	≥ 64	≥ 64	≤4	≥ 64	32	2	≥ 64	≥ 16	8	≤ 2	≤ 1	≤ 1	0.5	1	≤ 20	2
