# Supplementary material for: Regional surveillance of carbapenem-resistant Klebsiella pneumoniae responsible for bloodstream infections in Hebei province
Source: Front Cell Infect Microbiol. 2026 Aug 14;16:1883044. doi: 10.3389/fcimb.2026.1883044 (PMC13521795; doi:10.3389/fcimb.2026.1883044)
Supplement: Supplementary file 2 [file DataSheet2.pdf]

Supplementary Table 2

Isolate ID	SRA Accession (SRR)	Submission	BioProject	BioSample
K93	SRR39530553	SUB16272938	PRJNA1490004	SAMN61414734
K59	SRR39530577	SUB16272938	PRJNA1490004	SAMN61414712
K29	SRR39530605	SUB16272938	PRJNA1490004	SAMN61414687
K41	SRR39530592	SUB16272938	PRJNA1490004	SAMN61414699
K13	SRR39530620	SUB16272938	PRJNA1490004	SAMN61414673
K107	SRR39530538	SUB16272938	PRJNA1490004	SAMN61414748
K33	SRR39530601	SUB16272938	PRJNA1490004	SAMN61414691
K1	SRR39530623	SUB16272938	PRJNA1490004	SAMN61414662
K35	SRR39530598	SUB16272938	PRJNA1490004	SAMN61414693
K87	SRR39530559	SUB16272938	PRJNA1490004	SAMN61414729
K100	SRR39530546	SUB16272938	PRJNA1490004	SAMN61414741
K8	SRR39530567	SUB16272938	PRJNA1490004	SAMN61414668
K38	SRR39530595	SUB16272938	PRJNA1490004	SAMN61414696
K70	SRR39530570	SUB16272938	PRJNA1490004	SAMN61414719
K16	SRR39530618	SUB16272938	PRJNA1490004	SAMN61414675
K21	SRR39530614	SUB16272938	PRJNA1490004	SAMN61414679
K22	SRR39530613	SUB16272938	PRJNA1490004	SAMN61414680
K36	SRR39530597	SUB16272938	PRJNA1490004	SAMN61414694
K15	SRR39530619	SUB16272938	PRJNA1490004	SAMN61414674
K47	SRR39530585	SUB16272938	PRJNA1490004	SAMN61414705
K49	SRR39530583	SUB16272938	PRJNA1490004	SAMN61414707
K6	SRR39530589	SUB16272938	PRJNA1490004	SAMN61414666
K54	SRR39530581	SUB16272938	PRJNA1490004	SAMN61414709
K40	SRR39530593	SUB16272938	PRJNA1490004	SAMN61414698
K60	SRR39530576	SUB16272938	PRJNA1490004	SAMN61414713
K30	SRR39530604	SUB16272938	PRJNA1490004	SAMN61414688
K5	SRR39530600	SUB16272938	PRJNA1490004	SAMN61414665
K31	SRR39530603	SUB16272938	PRJNA1490004	SAMN61414689
K74	SRR39530568	SUB16272938	PRJNA1490004	SAMN61414721
K75	SRR39530566	SUB16272938	PRJNA1490004	SAMN61414722
k62-51	SRR39530535	SUB16272938	PRJNA1490004	SAMN61414751
K2	SRR39530622	SUB16272938	PRJNA1490004	SAMN61414663
K92	SRR39530554	SUB16272938	PRJNA1490004	SAMN61414733
K28	SRR39530606	SUB16272938	PRJNA1490004	SAMN61414686
K80	SRR39530563	SUB16272938	PRJNA1490004	SAMN61414725
K62	SRR39530574	SUB16272938	PRJNA1490004	SAMN61414715
K45	SRR39530587	SUB16272938	PRJNA1490004	SAMN61414703
K7	SRR39530578	SUB16272938	PRJNA1490004	SAMN61414667
K55	SRR39530580	SUB16272938	PRJNA1490004	SAMN61414710
K90	SRR39530555	SUB16272938	PRJNA1490004	SAMN61414732
K73	SRR39530569	SUB16272938	PRJNA1490004	SAMN61414720

K34	SRR39530599	SUB16272938	PRJNA1490004	SAMN61414692
K46	SRR39530586	SUB16272938	PRJNA1490004	SAMN61414704
K24	SRR39530610	SUB16272938	PRJNA1490004	SAMN61414682
K17	SRR39530617	SUB16272938	PRJNA1490004	SAMN61414676
K50	SRR39530582	SUB16272938	PRJNA1490004	SAMN61414708
K78	SRR39530564	SUB16272938	PRJNA1490004	SAMN61414724
K23	SRR39530612	SUB16272938	PRJNA1490004	SAMN61414681
K25	SRR39530609	SUB16272938	PRJNA1490004	SAMN61414683
K85	SRR39530561	SUB16272938	PRJNA1490004	SAMN61414727
K61	SRR39530575	SUB16272938	PRJNA1490004	SAMN61414714
K94	SRR39530552	SUB16272938	PRJNA1490004	SAMN61414735
K102	SRR39530543	SUB16272938	PRJNA1490004	SAMN61414743
K9	SRR39530556	SUB16272938	PRJNA1490004	SAMN61414669
K10	SRR39530545	SUB16272938	PRJNA1490004	SAMN61414670
K63	SRR39530573	SUB16272938	PRJNA1490004	SAMN61414716
K12	SRR39530621	SUB16272938	PRJNA1490004	SAMN61414672
K88	SRR39530558	SUB16272938	PRJNA1490004	SAMN61414730
K44	SRR39530588	SUB16272938	PRJNA1490004	SAMN61414702
K20	SRR39530615	SUB16272938	PRJNA1490004	SAMN61414678
K27	SRR39530607	SUB16272938	PRJNA1490004	SAMN61414685
k37-96	SRR39530530	SUB16272938	PRJNA1490004	SAMN61414755
K68	SRR39530571	SUB16272938	PRJNA1490004	SAMN61414718
K57	SRR39530579	SUB16272938	PRJNA1490004	SAMN61414711
K37	SRR39530596	SUB16272938	PRJNA1490004	SAMN61414695
K106	SRR39530539	SUB16272938	PRJNA1490004	SAMN61414747
K89	SRR39530557	SUB16272938	PRJNA1490004	SAMN61414731
K26	SRR39530608	SUB16272938	PRJNA1490004	SAMN61414684
K39	SRR39530594	SUB16272938	PRJNA1490004	SAMN61414697
K95	SRR39530551	SUB16272938	PRJNA1490004	SAMN61414736
K101	SRR39530544	SUB16272938	PRJNA1490004	SAMN61414742
K99	SRR39530547	SUB16272938	PRJNA1490004	SAMN61414740
k42-21	SRR39530531	SUB16272938	PRJNA1490004	SAMN61414754
K103	SRR39530542	SUB16272938	PRJNA1490004	SAMN61414744
K98	SRR39530548	SUB16272938	PRJNA1490004	SAMN61414739
K83	SRR39530562	SUB16272938	PRJNA1490004	SAMN61414726
K4	SRR39530611	SUB16272938	PRJNA1490004	SAMN61414664
K76	SRR39530565	SUB16272938	PRJNA1490004	SAMN61414723
K19	SRR39530616	SUB16272938	PRJNA1490004	SAMN61414677
K43	SRR39530590	SUB16272938	PRJNA1490004	SAMN61414701
K86	SRR39530560	SUB16272938	PRJNA1490004	SAMN61414728
K42	SRR39530591	SUB16272938	PRJNA1490004	SAMN61414700
K105	SRR39530540	SUB16272938	PRJNA1490004	SAMN61414746
K97	SRR39530549	SUB16272938	PRJNA1490004	SAMN61414738
K96	SRR39530550	SUB16272938	PRJNA1490004	SAMN61414737

k66-69	SRR39530533	SUB16272938	PRJNA1490004	SAMN61414752
k47-61	SRR39530529	SUB16272938	PRJNA1490004	SAMN61414756
K11	SRR39530534	SUB16272938	PRJNA1490004	SAMN61414671
k104-23	SRR39530525	SUB16272938	PRJNA1490004	SAMN61414760
K108	SRR39530537	SUB16272938	PRJNA1490004	SAMN61414749
k50-16	SRR39530528	SUB16272938	PRJNA1490004	SAMN61414757
K48	SRR39530584	SUB16272938	PRJNA1490004	SAMN61414706
k90-6	SRR39530524	SUB16272938	PRJNA1490004	SAMN61414761
k50-17	SRR39530527	SUB16272938	PRJNA1490004	SAMN61414758
K32	SRR39530602	SUB16272938	PRJNA1490004	SAMN61414690
k66-70	SRR39530532	SUB16272938	PRJNA1490004	SAMN61414753
K64	SRR39530572	SUB16272938	PRJNA1490004	SAMN61414717
K104	SRR39530541	SUB16272938	PRJNA1490004	SAMN61414745
K109	SRR39530536	SUB16272938	PRJNA1490004	SAMN61414750
k50-34	SRR39530526	SUB16272938	PRJNA1490004	SAMN61414759
